# The Dual-Path Analysis of the Effect of Difficult Nurse-Patient Relationship on Compassion Fatigue and Compassion Satisfaction in Nurses

**DOI:** 10.1097/jnr.0000000000000722

**Published:** 2026-01-09

**Authors:** Hui Ren, Tongshuang Yuan, Xin Yin, Leilei Liang, Junsong Fei, Xiaoying Liu, Chengbin Zheng, Huimin Wang, Jiaying Gao, Jiayuan Xu, Songli Mei, Hongyan Li

**Affiliations:** 1 Department of Nursing, The First Hospital of Jilin University, Changchun, China; 2 School of Public Health, Jilin University, Changchun, China; 3 The First Hospital of Jilin University, Changchun, China; † contributed equally

**Keywords:** affective empathy, compassion fatigue, compassion satisfaction, nurse-patient relationship, self-reflection

## Abstract

**Background:**

Although compassion fatigue has received significant attention in nursing management, compassion satisfaction has been less studied.

**Purpose:**

This study was designed to elucidate the positive and negative trends in nursing empathy from a dialectical perspective and investigate the impact of difficult nurse-patient relationship on compassion fatigue and satisfaction, and the underlying mechanisms.

**Methods:**

A cross-sectional study was conducted from November to December 2022 at a tertiary hospital in a northeastern province of China on 1,756 nurses. A moderated mediation model was constructed using structural equation modeling, and multi-group comparative analysis was used to examine the differences in the proposed model.

**Results:**

After adjusting for controlled variables, empathic concern and personal distress were shown to mediate the relationship between difficult nurse-patient relationship and, respectively, compassion satisfaction and burnout. Personal distress was found to mediate the relationship between difficult nurse-patient relationship and secondary traumatic stress, while self-reflection was found to moderate the effect of difficult nurse-patient relationship on empathic concern. Finally, differences in the structural model were detected between those with and without hospital traumatic experience.

**Conclusions/Implications for Practice:**

The findings reveal the mechanisms and boundary conditions underlying difficult nurse-patient relationship that influence compassion fatigue and compassion satisfaction. Interventions that increase the level of self-reflection and empathic concern may help reduce burnout and increase compassion satisfaction in nurses.

## Introduction

Exposure to various stressors from both patients and practice-related sources make the work environment of nurses unique (Woo et al., 2020). The results of prior research indicate nurses are more prone to compassion fatigue than other health care professionals (Cavanagh et al., 2019). Compassion fatigue, different from the general fatigue associated with increased job demands and job stress (Gander et al., 2011), is primarily associated with exposure to traumatic events and the emotional burden of others. This type of fatigue is marked by the loss of ability to manage emotional strain due to constant exposure to distressed individuals, ultimately resulting in emotional, physical, and spiritual depletion (Sacco et al., 2015). Compassion fatigue is common among nurses, psychologists, social workers, and others who are frequently exposed to the pain and difficulties of others (Gustafsson & Hemberg, 2022; Peters, 2018). In nursing practice, compassion fatigue is considered to be the end result of prolonged and close contact with patients, self-expenditure, and exposure to stress (Coetzee & Klopper, 2010).

Compassion fatigue is widely measured using the Professional Quality of Life Scale (ProQOL), developed by Stamm. This scale covers the three dimensions of secondary traumatic stress (STS), burnout, and compassion satisfaction. The first two (STS and burnout) are employed to measure negative trends in the workplace. STS refers to indirect exposure to the traumatic experiences or stressful situations of others (e.g., patients, victims) and stems from the desire to alleviate their suffering. STS generally manifests as fear, sleep disturbances, and avoidance behaviors. Burnout refers to fatigue, depersonalization, and reduced fulfillment due to prolonged exposure to occupational stressors. Conversely, compassion satisfaction is employed to measure a positive trend in the workplace which, in the context of nursing, captures the ability to implement effective health care services and help patients and their families (Galiana et al., 2022). The results of a literature review found level of compassion fatigue to increase over time in nurses and that nurses in Asia have the lowest levels of compassion satisfaction and most severe symptoms of compassion fatigue in the world (W. Q. Xie et al., 2021). Severe compassion fatigue is strongly associated with individual physical and psychological problems, potentially leading to substance abuse, patient avoidance, absentee behavior, and impaired quality of care (Sinclair et al., 2017), while compassion satisfaction is associated with positive effects on physical and mental health and work outcomes in nurses (Yu & Gui, 2022). Therefore, identifying both risk and protective factors for compassion fatigue and compassion satisfaction in nurses is essential in reducing adverse outcomes.

Previous research has confirmed compassion satisfaction to be a protective factor against compassion fatigue (Pérez-Chacón et al., 2021). Unfortunately, compassion satisfaction has not received sufficient attention in assessments of the consequences of empathy in nurses. A study by Ortega-Galan et al suggests moderate and high levels of compassion fatigue and moderate and high levels of compassion satisfaction may coexist (Ortega-Galan et al., 2020). Therefore, measuring nurses’ perceived quality of work in terms of both negative and positive aspects is necessary. In addition, ensuring health care providers find joy and meaning in their work is a key component of high-value health care service provision (Sikka et al., 2015). In this study, a dialectical perspective focusing on both compassion fatigue and compassion satisfaction in nurses is used to reveal the mechanisms underpinning their respective pathways.

### The Nurse-Patient Relationship (NPR)

The NPR, a work-oriented, caring interpersonal relationship that nurses develop with patients and their families, relates closely to quality of care and patient health outcomes and influences nursing performance and outcomes (Gou et al., 2022). The current NPR is strained (Xu & Fan, 2023). In addition, nurses frequently experience workplace violence. One meta-analysis found that nearly three-quarters (71%) of nurses in China had experienced workplace violence during the most recent 12-month period (Liu et al., 2022). The cyclical model of violence states that the inability to connect with patients, as well as NPR difficulties, increases the risk of workplace violence for nurses (Whittington & Wykes, 1994), which further exacerbates burnout and STS. In addition, according to the job demands resource model, continuous job demands lead to employees being unable to recover effectively from job-related stress, triggering a health-loss path leading to negative outcomes such as burnout (Demerouti et al., 2001). The NPR, one of the primary job requirements for nurses, requires nurses to invest a great deal of attention and energy to meet the needs of patients and their families. Consequently, nurses may experience physical and mental exhaustion, STS, and less compassion satisfaction. Based on the above, the authors hypothesize that difficult NPR will significantly and positively predict compassion fatigue and significantly and negatively predict compassion satisfaction in nurses.

### The Mediating Role of Affective Empathy

Empathy, a core aspect of high-quality health care, is also intimately linked to patient satisfaction as well as health care worker health outcomes, including burnout and emotion regulation abilities (Weisz & Cikara, 2021). Affective empathy refers to the ability of an individual to resonate strongly with the emotion of others and to generate spontaneous alternative emotional experiences. Emotions directed toward others represent empathic concern, while those directed inward represent personal distress (Davis, 1983). Under the comprehensive theoretical model of compassion fatigue, the demands and resource availability of patients elicit care provider stress-assessment processes, which trigger other-focus and self-focus, resulting in, respectively, empathic concern and personal distress. This ultimately results in, respectively, compassion satisfaction or compassion fatigue (Coetzee & Laschinger, 2018). Within poor NPR, patients may place excessive or even unscientific expectations and demands on nurses. Nurses will assess their individual resources and job resources to meet that demand. If nurses evaluate their resources as adequate, they usually do not perceive the difficult NPR as threatening and develop empathic concern. Thus, under adequate-resource scenarios, nurses actively connect with patients and care for them using compassionate care. The positive experiences of nurses caring for others increase. In contrast, when they assess their resources as inadequate, nurses experience personal distress, which is an aversive emotion associated with personal interests, as well as negative emotions such as fear and emotional vulnerability (Hayuni et al., 2019). Thus, under inadequate-resource scenarios, nurses choose to avoid or even isolate unpleasant experiences or stimuli, leading to compassion fatigue. In a previous study, higher personal distress was shown to be associated with higher compassion fatigue and lower compassion satisfaction levels (Stefan & Cheie, 2022). On this basis, we propose a hypothesis that difficult NPR affects both compassion fatigue and compassion satisfaction in nurses by triggering different types of affective empathy, including empathic concern and personal distress.

### The Moderating Role of Self-Reflection

Self-reflection is the self-examination and evaluation of one’s own thoughts, feelings, and behaviors. Individuals with good self-reflection have a clear understanding of themselves and make purposeful, targeted changes accordingly (Son, 2018). Eng and Pai found that self-reflective skills positively predict coping behaviors and clinical care competencies in medical students and negatively predict their stress levels (Eng & Pai, 2015). According to social cognitive theory, the interaction between personal and environmental factors influences individual behavior and development (Bandura & Cervone, 1983). From this perspective, an individual’s behavior and development are shaped by the external environment and driven by personal factors (including cognitive and emotional factors). Affective empathy, as an emotion regulation process, may be jointly influenced by difficult NPR and self-reflection ability. The ability to self-reflect is not merely the result of cognitive processes but also intricately linked to emotion management. Consequently, when confronted with difficult NPR (environmental factors), self-reflection enables individuals to gain a clearer perception and adjust their inappropriate responses within this context to reduce the incidence of personal distress. Furthermore, self-reflection assists individuals in managing their emotions effectively and enables them to better control their emotional output, averting personal distress stemming from excessive involvement. Also, through self-reflection, individuals can identify the factors that contribute to distress, fostering improved communication and understanding. This, in turn, enhances their capacity for empathetic concern. The existing research confirms the moderating effect of self-reflection in the relationship between self-compassion and social anxiety (Stefan & Cheie, 2022). Therefore, the hypothesis posited in this study is that self-reflection attenuates the negative predictive effect of difficult NPR on empathic concern and the positive predictive effect on personal distress.

### The Moderated Mediation Model

The adverse impacts of difficult NPR have become increasingly evident. However, most studies in the literature have measured NPR from the patient perspective. Moreover, nursing perspectives on this issue in the Chinese context are inadequate in the literature. In addition, few studies have examined the relationship between difficult NPR and compassion fatigue and compassion satisfaction in nurses. Therefore, increasing understanding regarding “whether” and “how” difficult NPR affects compassion fatigue and compassion satisfaction in nurses is important. Empathy, which is crucial to the delivery of high-quality care by nurses, is inherently tied to the practice of nursing (Bowden et al., 2023). As different types of empathy may predict different outcomes, existing studies, which tend to link empathy to compassion fatigue, are one-sided and inaccurate. In this study, empathic concern and personal distress were utilized as mediating variables, and self-reflection was introduced as a moderating variable to establish a moderated mediation model aimed at exploring the dual-path mechanisms through which difficult NPR influences compassion fatigue and compassion satisfaction in nurses. In addition, a history of trauma may be another factor of influence on compassion fatigue, as prior research has pointed to the negative impact on individuals of having a history of trauma. For example, Segal and Kagan found a significant association between having had traumatic experiences and higher levels of burnout and STS in midwives (Segal & Kagan, 2024). Conversely, traumatic events may also act as a protective factor that enhances an individual’s coping skills and resilience (Finzi-Dottan & Kormosh, 2016). Because studies in the literature have not provided evidence related to the effect of traumatic experiences on self-reflection, affective empathy, compassion fatigue and satisfaction, this study has further examined whether the moderated mediation model reveals differences between groups with and without hospital traumatic experience.

## Methods

### Study Design and Participants

This study was conducted at a tertiary hospital in a northeastern province of China from November to December 2022. The targeted hospital is the highest level of general hospital, with over 500 beds. After obtaining permission from the vice president for nursing and the director of the nursing department, the investigation was conducted online via the “Questionnaire Star” online questionnaire platform. The head of nurses distributed the electronic questionnaires in a WeChat group on social media, and all of the participants tapped the online voluntary informed consent button to access and answer the questionnaires. Any nurse working for more than one year as a clinical nurse under their current job title was invited to participate. A total of 1,868 nurses answered the questionnaire and a final sample of 1,756 was used for data analysis after excluding questionnaires associated with overly short or long response times, different responses on repeated items, or regular/repetitious responses. The study was carried out in accordance with the 1989 revision of the Helsinki Declaration and was approved by the Ethics Committee of the School of Public Health, Jilin University (Approval No. 2019-05-07). The purpose, procedures, and anonymity and information confidentiality policies of this study were explained on the first page of the questionnaire. Participation was voluntary and optional for all participants.

### Measurements

#### Demographic Information

Basic information collected from the participants included gender, age, marital status, educational level, department, professional title, working years, and working time (hours/week).

#### The Nurse-Patient Relationship

Nurse-perceived difficulty in communicating with patients was measured using the Difficulty in Doctor-Patient Relationship Questionnaire-8 (DDPRQ-8) scale. This scale has shown good validity and reliability (Azzez et al., 2019) and is currently widely used to measure the interaction and level of trust in the relationship between doctors and nurses and their patients and patient families. The original DDPRQ-8 scale contained three dimensions. In this study, only the three items in the dimension of “symptoms that showed the greatest correlation with the scale’s total score combine objective behavioral problems of patients and subjective feelings of medical staff” were used. The questionnaire items are all scored on a 5-point Likert scale, with 1 representing “*strongly agree*” and 5 representing “*strongly disagree*” and higher total scores indicating poorer NPR. In this study, the Cronbach’s alpha coefficient of the DDPRQ-8 was .71.

#### Empathy

Empathy was assessed using the 11-item affective empathy sub-dimension of the Chinese version of the Interpersonal Reactivity Index, which has been widely used among Chinese nurses (Shi et al., 2023). The affective empathy sub-dimension is distinguished into the two subscales of empathic concern and personal distress. Items are scored on a 5-point Likert scale ranging from 0 (*inappropriate*) to 4 (*very appropriate*). In this study, the Cronbach's coefficients for the two sub-dimensions were .80 and .83, respectively.

#### Compassion Fatigue and Compassion Satisfaction

In this study, the modified Chinese version of the ProQOL scale was adapted to measure compassion fatigue and compassion satisfaction in the participants (Yu et al., 2021). The ProQOL scale covers the three dimensions of compassion satisfaction, burnout, and STS, and each dimension contains 10 items scored on a 5-point Likert scale ranging from 1 (*inappropriate*) to 5 (*very appropriate*). The scale has previously shown good reliability and validity and is the most widely used instrument for measuring compassion fatigue in caregivers (Li et al., 2022). In this study, the Cronbach’s alpha coefficients for the three dimensions were .95, .86, and .87, respectively.

#### Self-Reflection

The Chinese version of the Self-Reflection Engagement subscale, originally developed by Grant et al. (2002), was used to measure self-reflection tendency. This subscale consists of five items all scored using a 7-point Likert scale ranging from 1 (*strongly disagree*) to 7 (*strongly agree*). The Cronbach’s alpha coefficient was .77 in this study.

#### Hospital Traumatic Experience

Hospital traumatic experience was assessed using the single question “Have you participated in a traumatic event while working in the hospital (e.g., earthquake rescue, epidemic rescue, rescuing a trauma patient of similar age/appearance to yourself/family member/friend, another event that caused serious physical and/or psychological damage to yourself or others, including family members and friends)?” The answer to this question was either “yes” and “no.”

### Data Analyses

Descriptive analysis was used to describe sample characteristics. Values of skewness and kurtosis were used to examine the normal distribution of data; *T* test and one-way analysis of variance were conducted to compare sociodemographic *and* work variable-based differences in compassion fatigue and compassion satisfaction; Pearson’s correlation coefficients were used to examine the association between studied variables; and the structural equation model (SEM) was used to test the mediating and moderating variables. All of the variables were processed and converted into *Z*-scores. A 95% deviation-corrected confidence interval (CI) was implemented based on 5,000 bootstrap samples, with effects considered to be statistically significant when the 95% CI did not include zero. Lastly, two nested models (i.e., free estimated and constrained) were constructed to explore the difference in the above dual-path model with regard to participants who had and had not had a traumatic experience in the hospital. All of the demographic variables were controlled in the mediation and moderated mediation analyses.

Indicators used to evaluate the fit of the hypothesized model with the data included: the ratio of chi-square statistics to degrees of freedom (χ^2^/*df*), Normed Fit Index (NFI), Türker-Lewis Index (TLI), Comparative Fit Index (CFI), and Root Mean Square Error of Approximation (RMSEA). TLI and CFI values of .90 and above were used to indicate an acceptable model, and RMSEA values <.08 and χ^2^/*df* values of 3 and below were used to indicate close model fit. Analyses were conducted in IBM SPSS Statistics 24.0 (IBM Corp., Armonk, NY, USA) and AMOS 24.0 statistical software. A two-tailed *p*-value < .05 was set for statistical significance.

## Results

### Sociodemographic Characteristics and Dimensions of ProQOL

The demographic information and the score distribution across categories of participants are presented in Table 1. The average age of participants was 35.14 (standard deviation, *SD* = 6.14) years, with 66.1% aged between 31 and 40 years and females accounting for 92.1%. Also, three-quarters (76.7%) were married and 89.5% had obtained a bachelor degree. The largest percentage of participants (27.6%) worked in the surgery department. The distribution of compassion satisfaction scores among variables including age, marital status, educational level, department, professional title, working years, and working time was significant (*p* < .01). Level of burnout differed significantly across gender, age, educational level, department, professional title, working years, and working time (*p* < .05), and mean STS score differed significantly across gender, age, department, professional title, working years, and working time (*p* < .05).

**Table 1:** Sample Demographics and the Distributions of the Professional Quality of Life Dimensions in Categorical Items (*N*=1,756)

Variable	*n* (%)	Compassion Satisfaction *M* (*SD*)	Burnout *M* (*SD*)	Secondary Traumatic Stress *M* (*SD*)
Gender				
Male	138 (7.9)	33.00 (8.49)	25.12 (7.61)	22.77 (7.49)
Female	1618 (92.1)	34.39 (9.00)	22.11 (7.39)	20.30 (6.52)
*t*		−1.752	4.590	3.743
*p*		.080	< .001	< .001
Age (years)				
≤30	349 (19.9)	33.78 (8.58)	22.05 (7.38)	19.75 (6.30)
31–40	1162 (66.1)	33.77 (9.05)	22.88 (7.52)	21.04 (6.74)
>40	245 (14.0)	37.44 (8.48)	20.22 (6.80)	18.99 (6.24)
*F*		17.995	13.532	12.625
*p*		< .001	< .001	< .001
Marital status				
Married	1346 (76.7)	34.73 (8.95)	22.21 (7.30)	20.70 (6.70)
Unmarried	373 (21.2)	32.62 (8.81)	22.71 (7.85)	19.76 (6.37)
Divorce/widowhood	37 (2.1)	34.84 (9.36)	23.54 (8.43)	20.46 (6.50)
*F*		8.194	1.122	2.931
*p*		< .001	.326	.054
Educational level				
Junior college or below	101 (5.8)	32.54 (8.87)	22.81 (7.79)	20.02 (6.21)
Bachelor degree	1572 (89.5)	34.22 (8.96)	22.44 (7.44)	20.59 (6.70)
Master's degree or above	83 (4.7)	37.52 (8.43)	20.04 (6.89)	19.42 (5.85)
*F*		7.386	4.329	1.494
*p*		.001	.013	.225
Department				
Internal medicine	360 (20.5)	34.35 (9.25)	21.84 (7.36)	19.69 (5.67)
Surgery	484 (27.6)	35.32 (8.74)	21.44 (7.20)	19.82 (6.40)
Obstetrics and gynecology	74 (4.2)	36.92 (8.29)	20.74 (7.06)	19.73 (6.11)
Pediatrics	243 (13.8)	35.29 (8.81)	21.67 (7.16)	20.87 (7.31)
Intensive care unit	265 (15.1)	32.01 (8.63)	24.45 (7.56)	21.63 (6.93)
Outpatient and emergency	117 (6.7)	30.93 (8.59)	25.41 (7.48)	22.75 (6.76)
Operating room	135 (7.7)	33.23 (9.15)	23.75 (7.32)	21.87 (7.58)
Others	78 (4.4)	36.51 (8.67)	19.73 (7.35)	18.40 (5.72)
*F*		8.219	10.306	6.868
*p*		< .001	< .001	< .001
Professional title				
Nurse	297 (16.9)	33.36 (9.20)	22.94 (7.73)	20.80 (6.56)
Senior nurse	556 (31.7)	33.59 (8.76)	22.62 (7.50)	20.61 (6.97)
Supervisor nurse	847 (48.2)	34.65 (8.90)	22.23 (7.26)	20.50 (6.50)
Co-chief nurse or above	56 (3.2)	40.57 (8.00)	18.21 (6.93)	17.84 (5.12)
*F*		12.021	6.766	3.266
*p*		< .001	< .001	.021
Working years				
≤4	239 (13.6)	34.30 (8.68)	21.69 (7.43)	19.45 (5.95)
5–9	478 (27.2)	33.10 (8.82)	23.05 (7.40)	21.07 (6.90)
10–14	707 (40.3)	33.69 (9.09)	22.98 (7.50)	20.89 (6.76)
≥15	332 (18.9)	37.23 (8.51)	20.46 (7.05)	19.59 (6.28)
*F*		16.183	11.064	6.125
*p*		< .001	< .001	< .001
Working time (hr/week)				
≤40	265 (15.1)	35.82 (8.37)	20.81 (6.61)	19.48 (6.30)
41–45	852 (48.5)	34.29 (9.01)	22.04 (7.29)	20.24 (6.48)
>45	639 (36.4)	33.64 (9.08)	23.40 (7.83)	21.26 (6.89)
*F*		5.531	12.892	8.025
*p*		.004	< .001	< .001

### Validity Tests for Variables

In this study, the average variance extracted (AVE) and confirmatory factor analysis (CFA) were employed to test the validity of the model. AVE values for all of the constructs ranged from .41 to .64. Although some AVE values fell below .5, previous studies suggest values greater than .4 are acceptable. CFA was conducted on the seven core variables using AMOS 24.0, with the results shown in Table 2. Relative to the other six models, the fit indicators of the seven-factor model (χ^2^/*df* = 4.337, RMSEA = .044, CFI = .945, TLI = .932, and NFI = .930) were significantly optimal, suggesting the variables of the model in this study have good discriminant validity.

**Table 2:** Confirmatory Factor Analysis Results

Model	χ^2^/*df*	RMSEA	CFI	TLI	NFI
Seven-factor model	4.337	.044	.945	.932	.930
Six-factor model	5.093	.694	.932	.917	.917
Five-factor model	6.970	.715	.900	.879	.886
Four-factor model	8.090	.063	.881	.856	.867
Three-factor model	8.963	.067	.866	.839	.852
Two-factor model	10.554	.074	.839	.806	.826
Single-factor model	13.099	.083	.796	.755	.784

*Note.* χ^2^/*df* = the ratio of chi-square statistics to degrees of freedom; RMSEA = Root Mean Square Error of Approximation; CFI = Comparative Fit Index; TLI = Türker-Lewis Index; NFI = Normed Fit Index.

### Common Method Bias Test

In this study, the Harman single-factor test was employed for common method biases testing (Podsakoff et al., 2003), with the results extracting 9 factors with eigenvalues greater than 1. The 25.96% variance explained by the first factor, which is below the cutoff value of .50, indicates the common method bias of the data to be below the threshold of concern.

### Correlation Analysis Among Key Study Variables

The kurtosis of the variables in this study ranged from −.54 to .90 and skewness ranged from −.96 to .74, indicating normal data distribution (Kim, 2013). The means and *SD*s, as well as the correlation analysis results for the key study variables, are presented in Table 3. With the exception of the nonsignificant relationship identified between empathic concern and personal distress, all of the key variables demonstrated statistically significant correlations, with coefficients between −.511 and .638 (*p* < .01). Difficult NPR and personal distress were associated positively with burnout and STS (*p* < .001) and negatively with compassion satisfaction (*p* < .001). Empathic concern and self-reflection were associated negatively with burnout and STS (*p* < .001) and positively with compassion satisfaction (*p* < .001).

**Table 3:** Descriptive Statistics and Correlations among Primary Study Variables

Variable	1	2	3	4	5	6	7
*M*	7.37	18.59	7.28	34.28	22.35	20.50	24.75
*SD*	2.60	4.02	4.51	8.97	7.45	6.63	5.32
Correlations							
1. Difficult NPR	1						
2. Empathic concern	−.211^***^	1					
3. Personal distress	.377^***^	.021	1				
4. Compassion satisfaction	−.407^***^	.383^***^	−.159^***^	1			
5. Burnout	.515^***^	−.214^***^	.341^***^	−.511^***^	1		
6. Secondary traumatic stress	.491^***^	−.112^***^	.437^***^	−.180^***^	.638^***^	1	
7. Self-reflection	−.209^***^	.304^***^	−.062^**^	.355^***^	−.190^***^	−.126^***^	1

*Note.* NPR = nurse-patient relationship; secondary traumatic stress and burnout reflect compassion fatigue.

^⁎⁎^ *p* < .01. ^⁎⁎⁎^ *p* < .001.

### Mediation Effect Analysis

An SEM was conducted to confirm the dual-pathway mechanism by which difficult NPR acts on compassion fatigue and compassion satisfaction, respectively. The model was shown to fit the data adequately (χ^*2*^ */df* = 2.753, *p* < .001, NFI = .988, TLI = .978, CFI = .992, RMSEA = .032). Difficult NPR was associated negatively with compassion satisfaction (β = −0.397, *p* < .001) and positively with burnout (β = 0.508, *p* < .001) and STS (β = 0.486, *p* < .001). Also, difficult NPR was associated negatively with empathic concern (β = −0.211, *p* < .001), while empathic concern was associated positively with compassion satisfaction (β = 0.310, *p* < .001) and negatively with burnout (β = −0.123, *p* < .001). However, empathic concern was shown to have no significant effect on STS (*p* > .05). The results of the mediating effects test are presented in Table 4. The mediating effect values of empathic concern between difficult NPR and, respectively, compassion satisfaction and burnout were −0.065 (95% CI = [−0.083, −0.050]) and 0.026 (95% CI = [0.016, 0.038]). The 95% CI did not contain zero, indicating the mediating effects as significant. However, the mediating effect of empathic concern between difficult NPR and STS was revealed to be insignificant (*p* > .05). In addition, difficult NPR was shown to be associated positively with personal distress (β = 0.337, *p* < .001), while personal distress was found to associate negatively with compassion satisfaction (β = −0.061, *p* < .01) and positively with burnout (β = 0.206, *p* < .001) and STS (β = 0.312, *p* < .001). The mediating effect values for personal distress between difficult NPR and, respectively, compassion satisfaction, burnout, and STS were −0.021 (95% CI = [−0.036, −.005]), 0.070 (95% CI = [0.055, 0.088]), and 0.105 (95% CI = [0.087, 0.125]). None of their 95% CI values contained 0, indicating the mediating effects to be significant.

**Table 4:** Bootstrap Confidence Interval and Effect Size of the Mediation Model

Mediation Path	β	Boot *SE*	Boot LL (CI)	Boot UL (CI)	*p*
Difficult NPR → Empathic concern → CS	−0.065	0.008	−0.083	−0.050	< .001
Difficult NPR → Empathic concern → Burnout	0.026	0.006	0.016	0.038	< .001
Difficult NPR → Empathic concern → STS	0.008	0.005	−0.001	0.017	.070
Difficult NPR → Personal distress → CS	−0.021	0.008	−0.036	−0.005	< .010
Difficult NPR → Personal distress → Burnout	0.070	0.008	0.055	0.088	< .001
Difficult NPR → Personal distress → STS	0.105	0.010	0.087	0.125	< .001

*Note.* NPR = nurse-patient relationship; CS = compassion satisfaction; STS = secondary traumatic stress; β = standard regression coefficient; Boot *SE* = bootstrap standard error; LL (CI) = the lower limit of 95% confidence interval; UL (CI) = the upper limit of 95% confidence interval; Secondary traumatic stress and burnout reflect compassion fatigue; The variables of gender, age, marital status, educational level, department, professional title, working years, and working time were all controlled.

### Moderated Mediation Effect Analysis

**FIgure 1:**
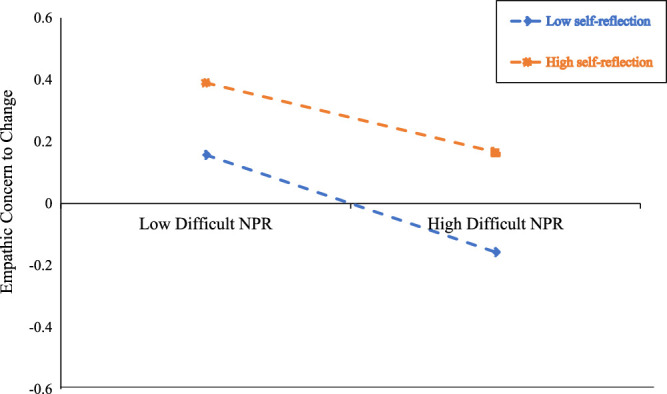
The Moderating Effect of Self-Reflection on the Relationship Between Difficult Nurse-Patient Relationship (NPR) and Empathic Concern

The results showed self-reflection to significantly moderate the relationship between difficult NPR and empathic concern (β = 0.049, *p* < 0.05), with a 95% CI of [0.004, 0.088], but not between difficult NPR and personal distress (β = 0.004, *p* > .05), with a 95% CI of [−0.038, 0.046], suggesting self-reflection may only be a predictive factor of empathic concern via difficult NPR. Simple slope tests were performed to further elucidate the moderating effect of self-reflection on the difficult NPR—empathic concern relationship, showing the interaction at 1 *SD* below the mean (*M* − 1*SD*) and 1 *SD* above the mean (*M* + 1*SD*) of self-reflection (Figure 1). For nurses with a low level of self-reflection, as the level of difficult NPR increased, empathic concern showed a significant decrease (β = −0.203, *p* < .001). For nurses with a higher level of self-reflection, as the level of difficult NPR increased, empathic concern exhibited a significantly decreasing and weaker trend (β = −0.112, *p* < .001). Therefore, the effect of difficult NPR on empathic concern weakened as the level of self-reflection increased. In addition, the bootstrapping method was used to test the moderated mediation model. The indirect conditional effect of difficult NPR on compassion satisfaction (β = −0.049, 95% CI = [−0.066, −0.034]) and burnout (β = 0.019, 95% CI = [0.011, 0.030]) through empathic concern were significant. However, the indirect conditional effect of difficult NPR on STS through empathic concern was insignificant (*p* > .05), and the effects of difficult NPR on compassion satisfaction and burnout through empathic concern were significant at low and high levels of self-reflection. The results suggest level of self-reflection influences the indirect relationship between difficult NPR and compassion satisfaction and burnout. Detailed information is given in Table 5.

**Table 5:** Conditional Indirect Effect of Difficult NPR on Compassion Satisfaction, Burnout and STS through Empathic Concern at Different Levels of Self-Reflection

Dependent Variable	Self-Reflection	β	Boot *SE*	Boot LL (CI)	Boot UL (CI)	*p*
CS	*M* − 1*SD*	−0.063	0.012	−0.087	−0.042	< .001
	*M*	−0.049	0.008	−0.066	−0.034	< .001
	*M* + 1*SD*	−0.035	0.009	−0.053	−0.018	< .001
Burnout	*M* - 1*SD*	0.025	0.006	0.014	0.040	< .001
	*M*	0.019	0.005	0.011	0.030	< .001
	*M* + 1*SD*	0.014	0.004	0.006	0.023	< .001
STS	*M* - 1*SD*	0.007	0.005	−0.001	0.017	.068
	*M*	0.006	0.003	0.000	0.013	.066
	*M* + 1*SD*	0.004	0.003	0.000	0.010	.055

*Note.* Secondary traumatic stress and burnout reflect compassion fatigue; The variables of gender, age, marital status, educational level, department, professional title, working years, and working time were all controlled; NPR = nurse-patient relationship; CS = compassion satisfaction; STS = secondary traumatic stress; *M* − 1*SD* = 1 *SD* below the mean; *M* + 1*SD* = 1 *SD* above the mean; β = standard regression coefficient; Boot *SE* = bootstrap standard error; LL (CI) = the lower limit of 95% confidence interval; UL (CI) = the upper limit of 95% confidence interval.

### Multiple-Group Path Model by Hospital Traumatic Experience

**Figure 2:**
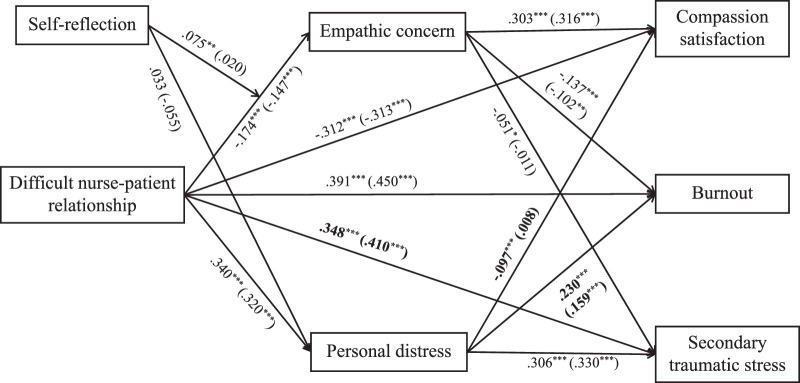
Standardized Estimation of the Moderating Mediation Model *Note*. Numbers in parentheses are the path coefficients in the model for the group without hospital trauma experience; The bolded coefficients indicate a significant difference between individuals with and without hospital trauma experience along that path; Secondary traumatic stress and burnout reflect compassion fatigue; All path coefficients were standard; The variables of gender, age, marital status, educational level, department, professional title, working years, and working time were all controlled. *p ≤ .05. **p < 0.01. ***p < .001.

A multi-group SEM was constructed to explore the differences in the proposed moderating mediation model. A good model fit of the unconstrained model was obtained (χ^*2*^ */df* = 2.608, *p* < .001, NFI = .962, TLI = .948, CFI = .976, RMSEA = .030), indicating the path model had a good fit for both groups. The path effects for both groups are presented in Figure 2. The χ^2^ difference between the two models was 93.632, which reached significance (*p* < .05), indicating the model performs unequally across hospital traumatic experience. After testing the difference in pathway coefficients between the two groups, the analysis results showed the value of critical ratio difference of the coefficients between the associations between difficult NPR and STS, personal distress and compassion satisfaction, and personal distress and burnout were all greater than 1.96, indicating the difference in the above paths coefficients across hospital traumatic experience were statistically significant. More specifically, the positive effect of difficult NPR on STS was statistically greater in the group without hospital traumatic experience (β = 0.410, *p* < .001) than the group with hospital traumatic experience (β = 0.348, *p* < .001). The positive effects of personal distress on burnout were statistically greater in the group with than the group without hospital traumatic experience (β = 0.230, *p* < .001 vs. β = 0.159, *p* < .001). Moreover, the significantly negative path was found only in the association between personal distress and compassion satisfaction in the group with hospital traumatic experience (β = −0.097, *p* < .001).

## Discussion

In this study, the dual-path mechanism and boundary conditions for the effect of difficult NPR on compassion fatigue and compassion satisfaction were analyzed and tested on a sample of nurses in China. This effort helps fill a gap in the current research on the issue of “whether” and “how” difficult NPR affects compassion fatigue and compassion satisfaction in nurses. The main findings of this study include: first, difficult NPR positively predicted compassion fatigue, but negatively predicted compassion satisfaction. Second, empathic concern and personal distress partially mediated the relationship between difficult NPR and both compassion fatigue and compassion satisfaction. Third, self-reflection negatively moderated the relationship between difficult NPR and empathic concern. Finally, the two clusters (nurses with and without hospital traumatic experience) differed significantly in the structural paths of the model and their coefficients.

The results show that difficult NPR, while positively influencing both burnout and STS, negatively influences compassion satisfaction. Lyu et al. (2023) also reported a positive correlation between difficult NPR and burnout syndrome. This correlation may be explained by the job demands resource model. The patient mistrust, anxiety, and complaints often associated with difficult NPR can exacerbate the self-perceived stress levels of nurses (Xu & Fan, 2023). The persistence of a discordant relationship between nurses and patients represents a drain on the physical and mental resources of nurses, and triggers a process of health depletion. In addition, the demanding attitudes of patients cause nurses to feel their resource investments do not achieve expected returns, promoting their adoption of avoidance behaviors in response. In summary, difficult NPR should be recognized as a significant source of burnout, STS, and decreased compassion satisfaction in nurses.

Also, difficult NPR was found to elicit different empathic outcomes through two different affective empathy pathways. Difficult NPR negatively predicted empathic concern, but positively predicted personal distress, which may be explained by the model of compassion fatigue. Difficult NPR results in the constant depletion of energy and resources in nurses. When nurses are already working with limited resources, difficult NPR become obstacles that lead to personal distress. Conversely, when nurses have access to abundant resources, empathic concern is bolstered. Because the nursing profession generally has access to limited work resources, difficult NPR usually leads to decreased levels of empathic concern and increased levels of personal distress. In addition, difficult NPR reflects patient dissatisfaction during medical visits as well as disharmony (e.g., mistrust and conflict) between medical staff and patients. Individuals with positive social contact experiences tend to report more empathic concern and less personal distress (Hechler et al., 2023), while negative nurse-patient interactions tend to decrease empathic concern and increase personal distress in nurses. Hechler et al. associated perceived threats to health, income, and social order with personal distress (Hechler et al., 2023). Difficult NPR enhances in nurses a sense of crisis in their work and health, thus increasing self-perceived levels of distress.

Notably, empathic concern negatively predicted burnout, but positively predicted compassion satisfaction, which is consistent with a previous study (Shi et al., 2023). Empathic concern reflects concern for the suffering of others and is accompanied by motivation to help. Thus, participants with high levels of empathic concern were less prone to burnout, as they sought ways to help their patients proactively. Moreover, they experienced higher levels of joy from helping and compassion satisfaction. However, the effect of empathic concern on STS was not significant in this study. This may be because STS relates more to an individual’s psychological resilience and the traumatic stress event, and thus coping ability may not be influenced by empathic concern. Personal distress was found to be positively associated with burnout and STS, but negatively associated with compassion satisfaction. These findings align with the existing evidence (Duarte et al., 2016). Personal distress elicited negative self-directed emotions that were accompanied by the desire of participants to withdraw or even escape from their current situation and their over-identification with the negative experience, which in turn demonstrated high burnout and STS levels and low compassion satisfaction levels.

Self-reflection was shown to offer potential protective effects, which aligns with a previous study (Tyler et al., 2022). Specifically, the negative effect of difficult NPR on empathic concern strengthened and weakened, respectively, at lower and higher levels of self-reflection. This finding is supported by social cognitive theory (Bandura & Cervone, 1983). Nurses develop a clear understanding of “what I should do” by reflecting on their work environment. Reflection motivates nurses to clarify their goals and provides motivation to achieve them. Therefore, when confronted with difficult NPR, nurses with a high degree of self-reflection are better able to adapt to changes in their environment, make adjustments and improvements, and safeguard their empathetic concern. On the contrary, nurses with low self-reflection lack the evaluation of their own thoughts, feelings, and behaviors, and are unable to make timely adjustments to their own state and behaviors when facing difficult NPR. Therefore, they are more likely to be negatively affected by difficult NPR, resulting in lower levels of empathic concern.

Another interesting finding of this study was that the positive predictive effect of difficult NPR on STS participants was lower in participants with a hospital traumatic experience than in their peers without. One reason for this may be that hospital traumatic experience give nurses experience in coping with emergency situations and increased self-efficacy and self-confidence. Thus, nurses are less likely to develop STS when they can analyze and deal with their own and their patients’ negative emotions objectively and calmly. This may also be due to the relatively higher age of nurses in the group with hospital traumatic experience. Increased age and clinical experience have been identified as protective factors against STS (W. Xie et al., 2020). Older individuals often have a better sense of control over emotional and stressful events and better professional performance and coping strategies in facing difficult NPR and personal distress (W. Xie et al., 2020). However, the relationship between demographic characteristics such as age, compassion fatigue, and compassion satisfaction has yet to be conclusively established (Jo et al., 2023). Notably, in the hospital traumatic experience group in this study, personal distress was a more significant positive predictor of burnout as well as a significant negative predictor of compassion satisfaction. This may be because nurses with hospital traumatic event experience are more emotionally empathetic, which would increase their emotional engagement and emotional burden. In addition, nurses with hospital traumatic event experience may be more reluctant to confide in others about their feelings and experiences and, as a result, fail to obtain adequate understanding and support. Prolonged emotional exhaustion and lack of social support may lead to nurses being more prone to psychological fatigue and burnout and to reduced joy in their work. However, due to the cross-sectional research design employed in this study, future longitudinal research designs and methods will be necessary to further clarify the relationship between hospital trauma event experience and, respectively, compassion satisfaction and compassion fatigue.

### Limitations and Future Research Directions

Several limitations of this study should be taken into consideration. First, the self-report data collection method used may introduce bias into the data. Future studies should combine evaluations from colleagues, supervisors, or other relevant individuals, objective evaluation indicators, and laboratory data. Second, to minimize the time spent completing the questionnaire, only three items in the dimension of “combination of objective behavior and subjective feelings” were used to assess the NPR. Although this dimension was previously confirmed to be most strongly correlated with the total score of the DDPRQ-8 scale, the full scale should be used in future studies. Third, other pathway mechanisms may exist between difficult NPR and, respectively, compassion fatigue and compassion satisfaction in nurses. Thus, a more comprehensive set of potential factors should be used, or further research into factors of influence should be conducted in future studies. Fourth, the cross-sectional research design used prevents the drawing of causal relationships among the study variables analyzed. Longitudinal design and models should be adopted in future research to examine the proposed moderated mediation model. Finally, the study sample was limited to one hospital in a northeastern province of China. Thus, the findings may not be generalizable to other populations. Nurses from multiple hospitals and a wider geographical region should be included in future studies.

### Relevance to Clinical Practice

This study has important implications for nursing management. The results underscore the importance of fostering good relationships between patients and nurses. Hospital managers should promote good communication and positive contact between nurses and patients through appropriate training courses, as well as provide nurses with more working resources, manageable working hours and task assignments, and other measures to improve the job satisfaction of nurses to indirectly improve NPR. In addition, hospital administrators should focus on developing nurses’ other-oriented affective empathy, potentially through interventions centered on promoting positive thinking. Finally, the importance of self-reflection should be emphasized in nurses’ work practices. Hospital managers should offer training courses on reflective skills and conduct diverse reflective activities to increase nurses interest in and commitment to reflection. Also, hospitals may deepen nurses’ understanding and awareness of their own thoughts, behaviors, and emotions in nursing work through the creation of work summaries and case sharing to help nurses better cope with stress and problems at work.

### Conclusions

The findings of this study show that difficult NPR significantly and positively influences compassion fatigue and negatively affects compassion satisfaction. Moreover, the findings confirm the existence of a dual-path way through which difficult NPR affects both compassion fatigue and compassion satisfaction. Difficult NPR is a positive predictor of burnout and a negative predictor of compassion satisfaction through empathic concern and personal distress, as well as a positive predictor of STS through personal distress. Finally, self-reflection was shown to negatively moderate the indirect effects of empathic concern. In summary, new evidence was provided in this study clarifying the relationship among the study variables, suggesting that while improving NPR, hospital administrators should also focus on promoting good workplace experiences for nurses in stressful situations by providing training in empathic concern and self-reflective skills to nurses.
